# Distractor Evoked Deviations of Saccade Trajectory Are Modulated by Fixation Activity in the Superior Colliculus: Computational and Behavioral Evidence

**DOI:** 10.1371/journal.pone.0116382

**Published:** 2014-12-31

**Authors:** Zhiguo Wang, Jan Theeuwes

**Affiliations:** 1 Center for Cognition and Brain Disorders, Hangzhou Normal University, Hangzhou, China; 2 Department of Cognitive Psychology, Vrije University, Amsterdam, The Netherlands; State University of New York Downstate Medical Center, United States of America

## Abstract

Previous studies have shown that saccades may deviate towards or away from task irrelevant visual distractors. This observation has been attributed to active suppression (inhibition) of the distractor location unfolding over time: early in time inhibition at the distractor location is incomplete causing deviation towards the distractor, while later in time when inhibition is complete the eyes deviate away from the distractor. In a recent computational study, Wang, Kruijne and Theeuwes proposed an alternative theory that the lateral interactions in the superior colliculus (SC), which are characterized by short-distance excitation and long-distance inhibition, are sufficient for generating both deviations towards and away from distractors. In the present study, we performed a meta-analysis of the literature, ran model simulations and conducted two behavioral experiments to further explore this unconventional theory. Confirming predictions generated by the model simulations, the behavioral experiments show that a) saccades deviate towards close distractors and away from remote distractors, and b) the amount of deviation depends on the strength of fixation activity in the SC, which can be manipulated by turning off the fixation stimulus before or after target onset (Experiment 1), or by varying the eccentricity of the target and distractor (Experiment 2).

## Introduction

**Figure 1:**
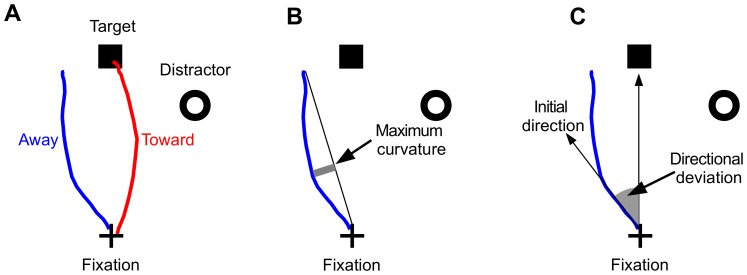
The distractor paradigm and measures for quantifying saccade trajectory deviations. (A) An illustration of the distractor paradigm with sample saccade trajectories deviating towards (red) and away from (blue) the distractor. (B) The “maximum curvature” measure of saccade trajectory deviation [45]. (C) The “directional deviation” measure of saccade trajectory deviation [46], see text for detail.

To deal with the abundance of visual information, at a given moment, an individual has to select only a small part of the environment by orienting his/her eyes to that part. This information sampling activity is facilitated by rapid eye movements, i.e., saccades. Saccades can be controlled in either bottom-up (stimulus-driven) or top-down (goal-driven) fashion. In the laboratory, the way bottom-up information affects the control of eye movements is usually explored with a simple paradigm that requires participants to make a saccade to a target while a visual onset distractor is simultaneously present, the so-called distractor paradigm. Previous studies using such paradigms have shown that the trajectory of a saccade may deviate towards or away from a task irrelevant distractor [1]–[4], see Fig. 1 for schematic illustrations of these observations and two methods for quantifying saccade trajectory deviations. Although extensive behavioral [5], [6], neurophysiological [7]–[9] and computational [1], [10]–[12] work has been done to reveal the underlying mechanism(s) of visual distractor induced deviations in saccade trajectory, no consensus has been reached in the field.

### The suppression theory of saccade deviation

Deviation in saccade trajectory was first regarded as evidence for the premotor theory of attention [13]–[16]. The dominant view in the literature now attributes saccade deviations to top-down suppression (inhibition) of the distractor location unfolding over time: initially insufficient suppression of the distractor location causes deviation towards and once full suppression is in place it causes deviation away from distractors [17]. For convenience, we will refer to this theory as the “suppression theory”. The suppression theory of saccade deviation is in agreement with several lines of empirical findings. First, the amount of deviation away from distractors increases with saccade latency [6], [18], [19], but see [20]. When saccade latency is short there is not enough time for the top-down suppression to develop and thus weak deviation away from or even deviation towards distractors is observed. Second, previous studies of the superior colliculus (SC), a layered midbrain structure that contains a topographic motor map that encodes the vector of saccades [21], have demonstrated that saccades deviate away from the response field of an inactivated SC region [11], [22], [23]. Third, McPeek and colleagues [7], [8] found that distractor evoked neuronal activity in the SC and the frontal eye fields (FEF) is stronger when saccades deviated towards than when saccades deviated away from distractors. These findings support the idea that insufficient suppression leads to deviation towards distractors and suppression developing later in time leads to deviation away from distractors.

Despite its popularity in the literature, the validity of the suppression theory was challenged by a recent single-unit recording study by White, Theeuwes and Munoz [9]. In this study, the authors presented distractors 400 ms before target onset, so as to allow sufficient time for the suppression at the distractor location to build up. The results showed that saccades robustly deviated away from the distractors even though there was no reduction of neuronal activity at SC sites representing the distractor location. Instead, there was a trend for stronger deviation away from distractors that evoked stronger neuronal activity, the exact opposite pattern of results predicted by the suppression theory. These observations suggest that the generally accepted view that top-down suppression causes saccade deviations towards and away from distractors has to be revised.

### The lateral interaction theory of saccade deviation

The lateral connection of neurons on the topographic motor map contained in the intermediate layers of the SC (SCi) is characterized by short distance excitation and long distance inhibition [24]–[26]. This lateral interaction can be captured by a neural field model [27], [28]. The neural field model has proven successful in simulating various saccade-related behavioral observations, including the global effect [29], [30] and trajectory deviations caused by lesion/inactivation [11] or visual distractor [10], [12]. It should be noted, however, that none of these computational studies has successfully simulated saccades deviating away from distractors. To fill up this gap, in a recent study [1], Wang, Kruijne and Theeuwes put forward a simple theory that claims that both deviation towards and away from visual distractors are caused by the lateral interactions in the SC. For convenience, we will refer to this theory as the “lateral interaction theory”.

**Figure 2:**
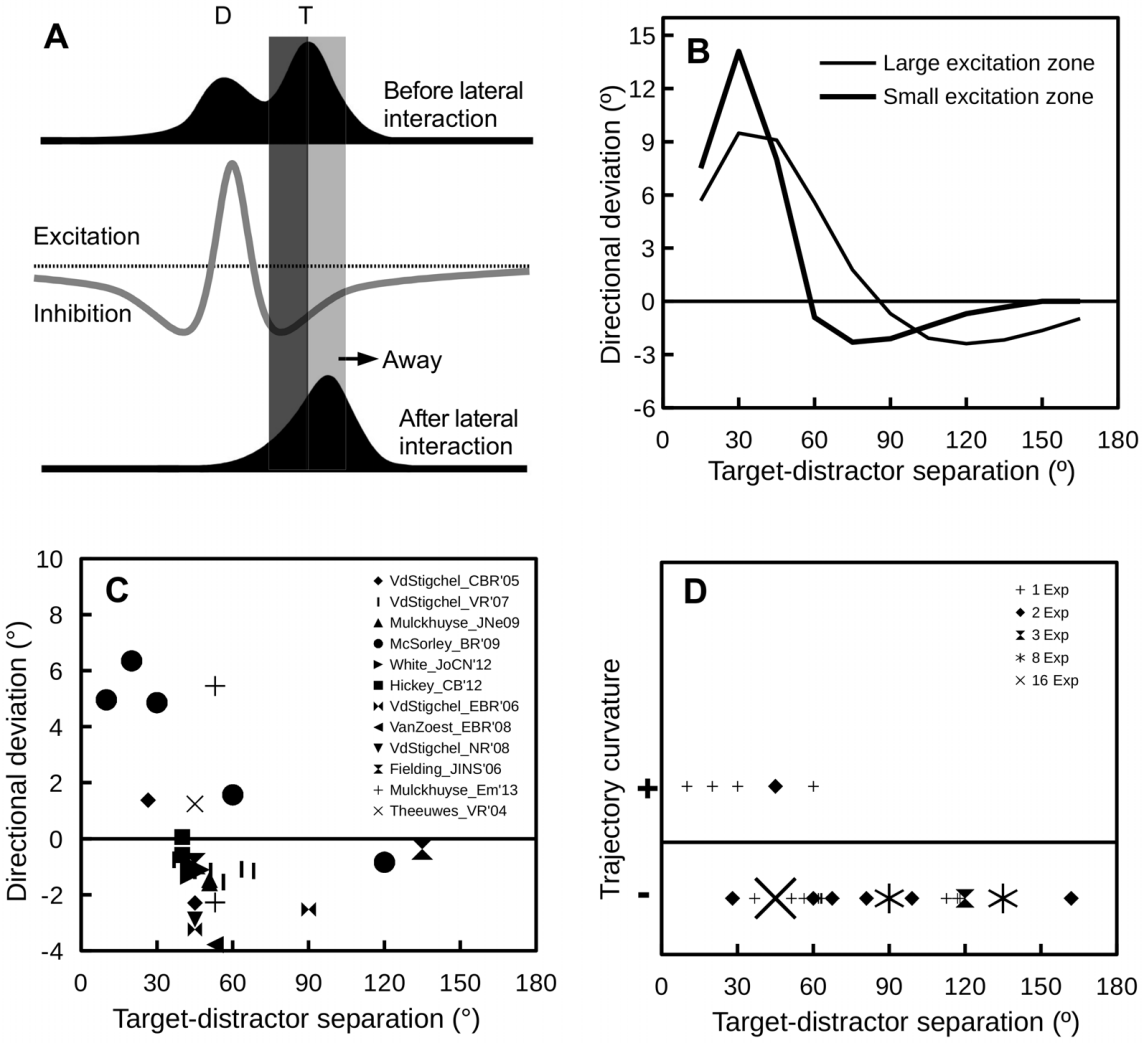
The lateral interaction theory and supporting evidence from the literature. (A) A schematic illustration of why the lateral interactions in the SC can produce saccades deviating away from the distractors (redrawn from [1]), see text for detail. (B) Simulation results of Wang et al. [1]. Positive and negative values on the y-axis denote deviation towards and away from distractors, respectively. Thick and thin lines denote simulations using kernels of small and large excitation zones, respectively. (C) A graphical meta-analysis of studies that reported directional deviations in saccade trajectory. In Theeuwes and Godijn [6], the saccade target and the distractor could appear at previously stimulated or unstimulated locations; only trials in which both the target and distractor appeared at unstimulated locations were considered. In Van der Stigchel and Theeuwes [52], saccade deviation was binned according to saccade latencies; the data reported here is the average over all latency bins. The same procedure was applied to the data from [6], [19], [49], [52]–[54]. The data from White et al. [9] was collected from two monkeys; the target-distractor SOA was manipulated in this study, only the 0-ms SOA condition was included here. (D) A graphical review of studies that reported saccade deviations quantified with trajectory curvature. Note that the curvature measures differed drastically across studies and thus only the direction of trajectory curvature was considered in this analysis, with “+” and “−” signs on the y-axis denoting trajectories curving towards and away from distractors, respectively. The size of the symbols represents the number of experiments contributing to each data point.

The critical component of the lateral interaction theory is the shape of the lateral interaction profile in the SC. While most in *vivo* studies of the lateral interactions in the SC have focused on the SCi [31], in *vitro* studies have demonstrated similar interactions in the superficial layers of the SC (SCs) [32]. In addition, it is well known that the neuronal activity in the SC is modulated by the inhibitory projection from the substantia nigra pars reticulata (SNr) [33]. So, the lateral interaction kernel derived from in *vivo* cell-recordings [34], [35] reflects the overall interplay between the SCi, SCs and the SNr, rather than only the synaptic connections within the SCi. Following previous modeling work [10], [34], [35], we assumed that the lateral interaction profile in the SC is Mexican-hat shaped. As shown in Fig. 2A (gray line), the lateral interaction profile has an excitation zone and two inhibition zones. In a distractor paradigm, when the distractor and the target are close in space, the distractor and target evoked activity “bubbles” will excite each other and merge into one activity bubble peaked at an SC site representing to a location in between these two stimuli. This will lead to saccade trajectories deviating towards the distractor. However, when the distractor and target are placed at a distance where they compete with each other, the target evoked activity bubble receives non-uniform inhibition from the distractor evoked activity bubble. That is, the side close to the distractor (shaded in dark gray in Fig. 2A) receives stronger inhibition than the other side (shaded in light gray in Fig. 2A). In the same vein, the distractor also receives non-uniform inhibition from the target. As a result, after lateral competition, the peak of the target activity (sustained by a top-down signal after the visual input dissipates) is pushed away, leading to saccade trajectories deviating away from distractors.

The simulation results of Wang et al. [1] are presented in Fig. 2B. As clearly shown in Fig. 2B, the model predicts that saccades deviate towards distractors that are close to the target and deviate away from distractors that are distal to the target. Due to lack of human neurophysiological data, the lateral interaction profile in the human SC is unknown. The simulation results using the lateral interaction kernel of Trappenberg et al. [35], which is based on monkey neurophysiology, is presented as a thin line, while the simulation results using a kernel with a much smaller excitation zone is presented as a thick line in Fig. 2B. The important message here is that the target-distractor separation at which the transition from deviation towards to deviation away occurs depends on the size of the excitation zone of the lateral interaction kernel.

### Graphical meta-analysis of the literature

To validate the model predictions of Wang et al. [1] and to identify empirically significant issues for further experimentation, a graphical meta-analysis of the literature was performed. By scanning the reference lists of three recent review articles [2]–[4] and searching Google Scholar for empirical papers that cited those review articles, we identified 54 relevant studies (as of Nov 18, 2013). A study was included in the meta-analysis if it had one or more experiments that met the following criteria: a) healthy subjects were tested or, in case of a patient study, healthy controls were tested; b) used the distractor task (see Fig. 1A for illustration); and c) the target and distractor were peripheral onsets and were presented simultaneously to the participants. Studies that used endogenous targets (i.e., an arrow at fixation, pointing to a predefined target location) [36]–[38] or had multiple distractors [39]–[42] were not considered. Furthermore, studies that explored saccade trajectory deviation using visual search tasks [7], [8], [43] or attention tasks [13]–[15], [17], [44] were also excluded from the analysis. The results of the meta-analyses, which were based on the remaining 27 empirical papers, are presented in Fig. 2C and 2D.

Before discussing the meta-analysis results, we would like to point out that in the literature, two groups of methods exist for quantifying deviation in saccade trajectories [2]. One group of methods emphasizes the curvature of saccade trajectories, that is, how much a saccade trajectory deviates from the direct path between the start and endpoint of a saccade [45], while other methods examines the (initial, final or average) direction of saccade trajectories relative to the direction of the saccade target [46]. For convenience, we will refer to these two groups of trajectory deviation measures as “trajectory curvature” and “directional deviation”, respectively.

Because it is easy to calculate, a trajectory curvature measure named “maximum curvature” [45] has been frequently used in the literature [5]. Maximum curvature is defined as the maximum distance of the sample points to the straight path between the start and end of a saccade trajectory (see Fig. 1B), sometimes further divided by the amplitude of the same saccade [18]. One often used directional deviation measure is “initial directional deviation”, defined as the direction of an early (e.g., the fifth) sample point of a saccade trajectory, relative to the target direction [46] (see Fig. 1C). Because Wang et al. [1] used a 1-dimensional neural field model that encoded only the saccade direction, their simulation results were about the directional deviation rather than the curvature of saccade trajectories.

Studies that examined directional deviations [6], [9], [19], [46]–[55] are summarized in Fig. 2C. A careful examination of Fig. 2C reveals several interesting observations. First, in agreement with the simulation results of Wang et al. [1], a transition from deviation towards distractors to deviation away from distractors emerges as the target-distractor angular separation increases. Second, the simulation results obtained using a kernel of a small excitation zone (thick line in Fig. 2B) seem to agree with behavioral findings more than those obtained using a kernel of a relatively large excitation zone (thin line in Fig. 2B). With a large excitation zone, the transition from deviation towards to deviation away occurs at a fairly large target-distractor separation (∼90°), while empirical results suggest that the transition occurs at a target-distractor separation of about 40°. Third, previous studies were mainly concerned with relatively small target-distractor separations. The simulation results of Wang et al. [1], which suggest that the amount of deviation away from distractors decreases as target-distractor separation increases, cannot be verified by simply synthesizing data available in the literature. To better characterize the relationship between saccade deviation and target-distractor separation with behavioral experiments, parametric manipulation of the target-distractor angular separation is needed. Finally, stimulus eccentricity (represented by the symbol size in Fig. 2C) seems to also play a role. Deviations in the “away” direction were generally weaker for more eccentric targets (and distractors).

For completeness, we also performed a graphical meta-analysis of studies that reported the curvature of saccade trajectories [5], [18], [20], [46], [48], [49], [56]–[67]. Because trajectory curvature measures varied drastically across studies, it is not possible to do a quantitative review (like the one presented in Fig. 2C), unless we reanalyze the raw data of all these studies. Thus, only the direction of trajectory curvature, i.e., towards (+) or away from (−) distractors, was considered in this analysis. The results are presented in Fig. 2D, with the size of symbols representing the number of experiments contributing to each data point. It is clear from Fig. 2D that, when measured with trajectory curvature, saccades also deviate towards close distractors and deviate away from remote distractors.

### Purpose of the present study

As shown in Fig. 2B, the amount of deviation towards distractors generated by the neural field model of Wang et al. [1] was significantly larger than those observed in previous behavioral studies (Fig. 2C). This is likely due to the fact that Wang et al. [1] did not consider the fixation activity at the rostral pole of the SC, which globally suppresses neuronal activity evoked by both the target and distractor. To address this issue, simulations considering the effect of SC fixation activity were first performed. Predictions generated by these simulations were then verified with behavioral experiments, in which both the strength of fixation activity and the angular distance between the target and the distractor were manipulated. The latter manipulation was included because, as clearly shown in Fig. 2C, large target-distractor distance (>60°) is largely unexplored in the literature.

## Simulations

As discussed above, one of limitations of Wang et al. [1] is that the fixation activity at the rostral pole of the SC was not considered in their simulations. To reveal the effect of fixation activity on saccade deviations, new simulations were performed using the same neural field model of the SC.

### Fixation activity and saccade deviation: A conceptual analysis

**Figure 3:**
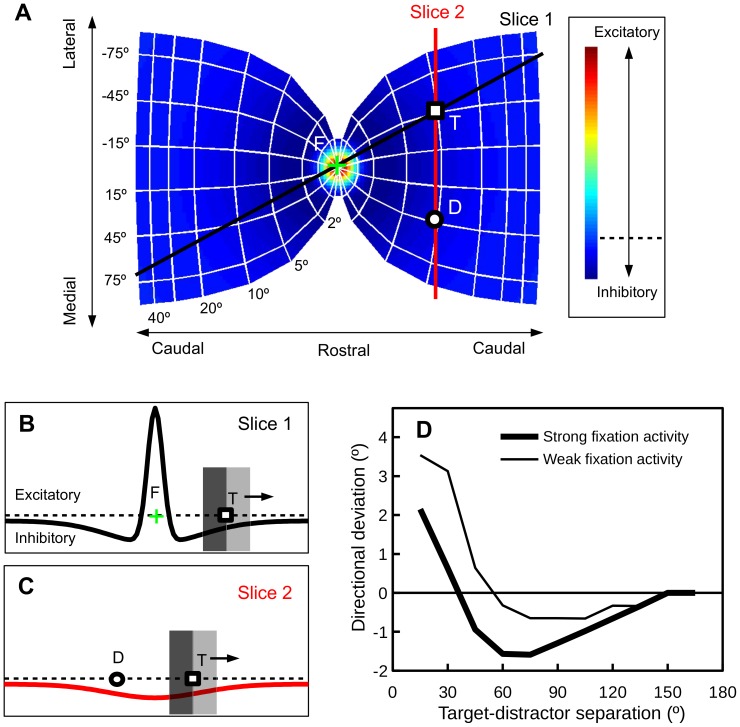
The strength of fixation activity modulates saccade trajectory deviation. (A) The motor map in the SC, drawn from the equations and parameters given by van Gisbergen, van Opstal and Tax [79]. The color gradient represents the strength of connections originating from the fixation zone (the rostral pole of the SC). T: Target; D: Distractor; F: Fixation. (B) Non-uniform inhibition from the fixation zone can cause the target-evoked activity to drift away from the fixation, see text for details. (C) The inhibition from the fixation zone can also cause the target-evoked activity to drift away from the distractor, see text for details. (D) Saccade deviations obtained from simulations in a neural field model of the SC [1], [35], assuming that the fixation activity was either weak or strong.

In a distractor paradigm, how saccade direction and amplitude are affected by fixation activity is graphically analyzed in Fig. 3. The color gradient in Fig. 3A represents the strength of connections originating from the rostral pole of the SC, i.e., the fixation zone. When appearing in the periphery, due to lateral inhibition, the target and distractor evoked activities receive inhibition from the fixation zone. The effect of this fixation-related inhibition is two-fold. First, because the lateral interaction profile is Mexican-hat shaped, as illustrated in Fig. 3B, the target evoked activity will drift away from the fixation zone (along Slice 1 in Fig. 3A). The stronger the fixation activity the further the drift, the larger the saccade amplitude. A second and more interesting effect of the fixation-related inhibition is illustrated in Fig. 3C. Because of the Mexican-hat shaped interaction profile, there exists a non-uniform inhibition along the path where the distractor and target interact (Slice 2 in Fig. 3A). As a result, saccades to the target will be pushed away from the distractor by the fixation activity. The stronger the fixation activity, the stronger the deviation away from the distractor. Importantly, as can be inferred from Fig. 3C, the effect of fixation-related inhibition on saccade deviation depends on the distance between the target and the distractor. When the target and the distractor are far apart, the strength of fixation activity should have little or no effect on saccade deviation.

### Model architecture

To demonstrate the idea that the strength of fixation activity modulates deviations in saccade direction, simulations were performed in a 1-dimensional neural field model of the SC [1]. The model, which has *n = 1000* nodes (leaky integrators), roughly represents 5-mm of SC tissue encoding the straight path through the target and distractor (see Fig. 3A). Equation 1 defines the connection strength (*w_ij_*) between two nodes. Because our graphical meta-analysis of the literature suggests that an interaction kernel with a small excitation zone could better predict human behavioral data (see Fig. 2B and 2C), the following parameters were used in our simulations: *a = 144, b = 24, c = 9*, *σ_a_ = 0.25 mm*, *σ_b_ = 1.5 mm*. The internal dynamics of a node is described in Equation 2, where *τ = 10 ms*. A sigmoid gain function is used to relate the discharge level of a node to its internal state, where *β = 0.08*.
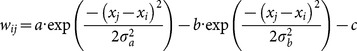
(1)

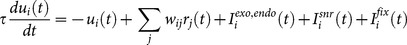
(2)

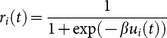
(3)


Nodes in the network receive both excitatory (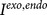) and inhibitory (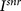 and 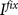) inputs. The excitatory input was further dissected into exogenous (visual) and endogenous (top-down) inputs [35], [68]. It should be noted that the endogenous input represents a top-down selection of the target and thus was only applied to the target location. The excitatory inputs were assumed to have a Gaussian spatial shape (see Equation 4). In our simulations, the width of the excitatory inputs were fixed at *σ_e_ = 0.53 mm*, close to estimations that are based on cell-recordings in the SC [24]. The temporal dynamics of the exogenous and endogenous inputs are described in Equations 5 and 6, respectively. As in previous modeling work [35], the exogenous input reached the SC 70 ms after stimulus onset (*t_on_*) while the endogenous input reached the SC 50 ms later, i.e., 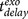 = *70* ms, 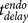 = *120* ms. For all simulations, the strength of exogenous and endogenous excitatory inputs were set to *e_exo_ = 30* and *e_endo_ = 15*, respectively. The exogenous input was transient and decayed exponentially (*τ = 10 ms*). The endogenous input was sustained and was turned off when the saccade initiation threshold (80% of the maximum discharge level) was crossed (*t_off_*). For simplicity, the inhibition from the substantia nigra pars reticulata (SNr) was fixed at 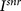 = *−16* for all nodes.
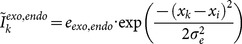
(4)

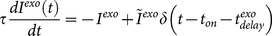
(5)

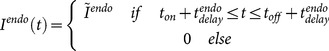
(6)


In our simulations, it was assumed that that target and distractor always appeared at locations mirroring the horizontal meridian. Thus, the inhibition coming from the fixation zone could be approximated with a sustained Gaussian-shaped inhibitory input (Equation 7), centered at the midpoint between the target and distractor (*x_c_*). The width of this input was set to *σ_fix_ = 0.7 mm*, while the strength of this input was set to either weak (*f = −0.5*) or strong (*f = −1.8*). Fixation neurons at the rostral pole of the SC are characterized by tonic discharge during active fixation and pause during saccades [69]. Thus, the inhibitory input coming from the fixation zone was applied to the model neurons from the beginning of each simulation trial and was turned off after the saccade initiation threshold was crossed (*t_off_*), see Equation 8.
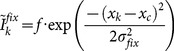
(7)

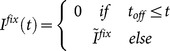
(8)


### Simulation results

The simulation results are presented in Fig. 3D. First and foremost, replicating previous findings [1], saccades deviated towards close distractors and away from remote distractors. Second, when inhibition from the fixation zone of the SC was considered, the amount of deviation towards distractors reduced to a range normally reported in previous empirical studies (about 1°–7°). Third, fixation activity modulated the amount of directional deviation, with strong fixation activity causing a shift in the “away” direction. Last but not least, as has been discussed previously, the modulatory effect of fixation activity on saccade deviation was weak (largely absent) for relatively large target-distractor separations. These simulation results provided us with testable predictions for further behavioral experimentation.

## Experiment 1: Gap vs. overlap

The primary purpose of this experiment was to verify the model predictions presented in the previous section (see Fig. 2D). To be more specific, directional deviations of saccade trajectory depends on both the target-distractor distance and the strength of fixation activity. To test these predictions, a within-subject design was adopted in which a) the target-distractor angular separation was varied between 15° and 150°, and b) the fixation stimulus was removed either 150 ms before target onset (gap) or 150 ms after target onset (overlap). The manipulation of temporal gap/overlap was introduced to vary the strength of fixation activity in the SC. Dorris and colleagues [70], [71] showed that temporal gaps can reduce fixation activity and this reduction of fixation activity reaches maximum at a gap duration of about 200 ms. Whether temporal overlaps increase fixation activity is currently unknown. However, for the present purpose, it is safe to infer that fixation activity was stronger in the overlap than in the gap condition.

### Materials and Methods

The human research protocol described here was approved by the local ethical committee at Vrije Universiteit and all participants gave written informed consent.

#### Participants

Sixteen university students (11 female, 5 male) participated this experiment for course credits or monetary compensation (9 €/hour). They all reported normal or corrected to normal vision and their mean age was 21 years.

#### Stimuli, apparatus and procedure

The saccade target was a filled gray square (1°×1°) and the distractor was a gray circle (d = 1°). All stimuli were presented against a black background on a 21 inch CRT monitor. The visible area of the monitor measured 39°×29.3° at a view distance of 61 cm. Stimulus presentation and data collection was controlled by a Windows computer running custom software written in Python. A video-based eye tracker (Eyelink II), with a spatial resolution of 0.2° or better, was used to monitor the participant's direction of gaze at a sampling rate of 500 Hz.

Self-paced drift correction was performed at the beginning of each trial, then a gray fixation cross (1°×1°) appeared at the center of the display. The target, which was presented 500–750 ms later, could appear at two possible locations 10° from the fixation cross in the upper visual field, 15° (polar angle) left or right to the vertical meridian. This design feature was adopted to reflect the fact that the simulations performed in Wang et al. [1] only considered oblique saccades. When a vertical saccade is executed, the evoked neuronal activity is shared on both colliculi, the exact location and shape of this distributed activity are unknown. Besides, the possible role of commissural SC projections in the coordination of the two SC is not fully understood [72]–[74]. By testing only oblique saccades, as in Wang et al. [1], the target and the distractor can both be represented in the same colliculus and this complication can be avoided. The distractor was absent on 20% of the trials. When presented, the distractor always appeared at the same eccentricity and in the same visual field (left or right) as the target. The angular separation between the distractor and the target could be 15°, 30°, 45°, 60°, 75°, 90°, 105°, 120°, 135°, or 150°. The fixation cross was turned off either 150 ms before (gap condition) or 150 ms after (overlap condition) target onset. It is important to keep in mind that target-distractor distance and temporal gap/overlap were intermixed within a block of trials in the present experiment.

The participants were tested with 3 blocks of 200 trials. A practice block of 24 trials was provided at the beginning of each experimental session. A trial was regarded as an error trial and a warning message was displayed if saccades were detected during fixation or the landing position of the critical saccade missed the target for more than 3° (visual angle). All error trials were later presented to the participant in a random order, until all trials were completed correctly.

#### Dependent measures of interest

Only the primary saccade following the presentation of the target was analyzed. In the present experiment, the primary measure of interest is the deviation of initial saccade direction. Initial saccade direction was estimated with the start point of a saccade and the gaze position 10 ms into the same saccade, see [52] for similar measures. Directional deviation was quantified as the difference in initial saccade direction between distractor present and absent trials, with positive and negative values denoting deviation towards and away from distractors, respectively.

As mentioned previously, a large chunk of the literature has been devoted to the exploration of saccade deviations quantified with trajectory curvature. So, here we also report a trajectory curvature measure of saccade deviation, i.e., maximum curvature [45]. Maximum curvature was defined as the maximum distance between the sample points to the straight path from the start to the endpoint of a saccade. Maximum curvature was referenced to the mean trajectory curvature of distractor absent trials, with positive and negative values denoting deviation towards and away from distractors, respectively. Ludwig and Gilchrist [45] compared several trajectory curvature measures and concluded that the quadratic curve fitting method was the best. Here we use maximum curvature because a) it is easy to compute, and b) Ludwig and Gilchrist [45] showed that maximum curvature is highly correlated with more sophisticated trajectory curvature measures, including the one derived from quadratic curve fitting.

Other dependent measures of interest are saccadic response time (SRT) and saccade amplitude. SRT was defined as the time interval between target presentation and saccade onset. Because the present experiment manipulated the temporal gap/overlap between fixation offset and target onset, SRTs were expected to be shorter in the gap than in the overlap condition, i.e., the gap effect [75], [76]. Saccade amplitude was also analyzed in the present study because, as briefly discussed previously, the lateral interaction theory predicts larger amplitudes for saccades in the overlap condition. Saccade amplitude was defined as the shortest distance between the start and endpoint of a saccade.

### Results

Due to technical issues, one participant (No. 14) completed partial (2/3) of the experimental trials. Careful off-line inspection of the eye movement data identified that the experimental script failed to recycle a small number of error trials (0.54%). These trials were excluded from the analyses. For successfully completed trials, those were excluded from the analyses if a) the SRT was shorter than 80 ms (6.16%) or longer than 500 ms (1.22%), b) the initial saccade direction deviated more than 30° from the target direction (2.64%), or c) the starting point of the primary saccade deviated more than 1.5° from the fixation cross (1.40%). After data cleansing, 88.91% of the trials remained.

**Figure 4:**
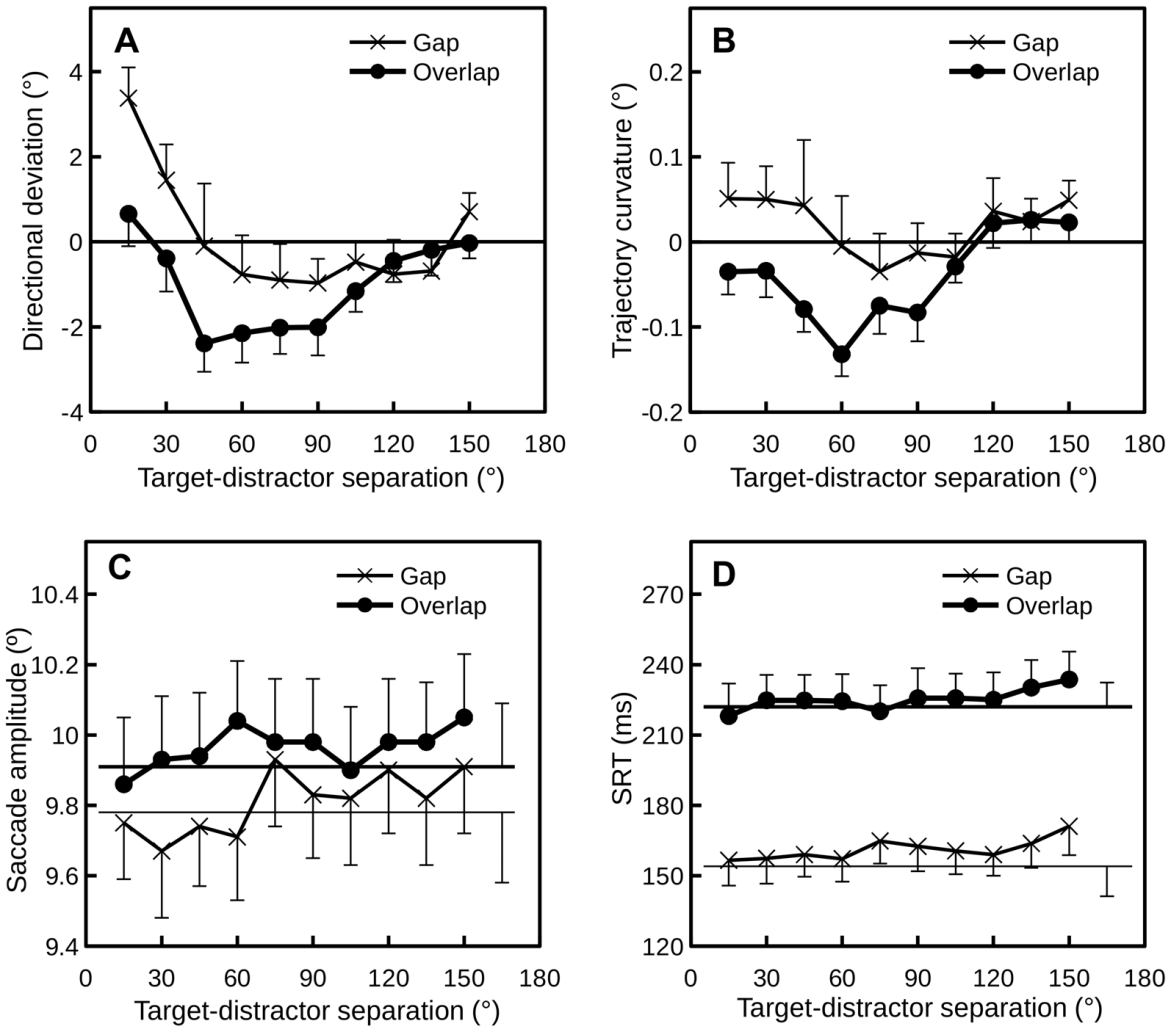
Mean directional deviation (A), trajectory curvature (B), saccade amplitude (C) and SRT (D) for each target-distractor separation in the gap and overlap conditions in Experiment 1. Error bars denote ±1 SE; for clarity, only one side of the error bars is drawn. In (C) and (D), thick and thin horizontal lines denote distractor-absent trials in the overlap and gap conditions, respectively.

The mean directional deviation, trajectory curvature, saccade amplitude and SRT of each experimental cell are presented in Fig. 4. Repeated measures ANOVAs of these measures, with variables condition (gap vs. overlap) and target-distractor separation (15°–150°), were performed and the results are presented below.

#### Directional deviation

An ANOVA of directional deviations revealed a significant main effect of target-distractor separation, F(9, 135) = 7.39, p<0.001, saccades deviated towards close distractors and deviated away from remote distractors (see Fig. 4A). The main effect of condition did not reach significance, F(1, 15) = 2.63, p = 0.13, however, a significant interaction between condition and target-distractor separation was observed, F(9, 135) = 2.17, p<0.05. Planned comparisons revealed reliable difference between the gap and overlap conditions when the target-distractor separation was 15°, t(15) = 4.18, p<0.01, and 30°, t(15) = 3.04, p<0.01.

#### Trajectory curvature

Analysis of trajectory curvature also revealed a main effect of target-distractor separation, F(9, 135) = 3.30, p<0.01. Though the main effect of condition did not reach significance, F(1, 15) = 2.17, p = 0.16, the interaction between condition and target-distractor separation reached marginal significance, F(9, 135) = 1.75, p = 0.08. Further analysis revealed reliable difference between the gap and overlap conditions when the target-distractor separation was 15°, t(15) = 1.95, p<0.07, 30°, t(15) = 2.70, p<0.01 and 60°, t(15) = 2.03, p = 0.06.

#### Saccade amplitude

Analysis of saccade amplitudes revealed significant main effects for condition, F(1, 15) = 9.63, p<0.01 and target-distractor separation, F(9, 135) = 3.67, p<0.001. The main effect of condition was observed because saccade amplitudes were generally larger in the overlap than in the gap condition (see Fig. 4C). The interaction between condition and target-distractor separation also reached significance, F(9, 135) = 1.95, p<0.05. Further analysis revealed that the amplitude difference between the gap and overlap conditions was statistically reliable when the target-distractor separation was 30°, 45°, 60°, 90°, 135° and 150°, all t>2.33 all p<0.05. As shown in Fig. 4C, when the distractor was not presented, the mean saccade amplitude of the overlap condition (thick horizontal line) was larger than that for the gap condition (thin horizontal line), t(15) = 2.04, p<0.05.

#### SRT

An ANOVA of the SRTs revealed significant main effects for condition, F(1, 15) = 226.7, p<0.001, and target-distractor separation, F(9, 135) = 3.32, p<0.01. The interaction between condition and target-distractor separation was not significant, F(9, 135) = 0.79, p = 0.63. As is clear from Fig. 4D, the main effect of condition occurred because SRTs in the gap condition were about 60 ms faster than those in the overlap condition. Note that, when no distractor was presented, SRTs in the gap condition (thin horizontal line) were still faster than those in the overlap condition (thick horizontal line), t(15) = 13.35, p<0.001, i.e., the gap effect. The main effect of target-distractor separation occurred because there was a trend for SRTs to increase with target-distractor separation, replicating the classic remote distractor effect [77].

### Discussion

Simulations using a neural field model of the SC showed that task irrelevant visual distractors can cause the direction of saccades to deviate towards or away from the distractors, depending on the angular distance between the target and the distractor [1]. The simulation results presented here suggest that the amount of saccade deviation also depends on the strength of fixation activity at the rostral pole of the SC (see Fig. 3D). To verify this prediction, a behavioral experiment manipulating the temporal gap/overlap between fixation offset and target onset was performed. The primary results of interest are those presented in Fig. 4A, i.e., deviations in initial saccade direction. It is clear that the behavioral results resemble those produced by the neural field model (Fig. 3D), supporting the lateral interaction theory of saccade deviation [1]. In addition to deviations in initial saccade direction, here we also report saccade deviations quantified with a trajectory curvature measure [5], [18]. As shown in Fig. 4B, when measured with trajectory curvature, the amount of deviation also varied with the manipulation of target-distractor separation and temporal gap/overlap.

One important finding of the present experiment is that saccade amplitudes were found to be larger in the overlap than in the gap condition (see Fig. 4C). This confirms a novel prediction of the lateral interaction theory. Because the lateral connection in the SC is Mexican-hat shaped, when the target appears in the periphery, the target evoked activity will be pushed away by the fixation activity (see Fig. 3B). As a result, the stronger the fixation activity, the larger the saccade amplitude. It is important to note that, although not statistically reliable, a similar pattern of results has been reported in an early study by Kingston and Klein [78]. This effect deserves further study.

In the literature, several researchers have reported that short-latency saccades are accompanied by deviation towards distractors and long-latency saccades are accompanied by deviation away from distractors [6], [18]. This observation has been regarded as evidence for the suppression theory because suppression at the distractor location takes time to develop. As shown in Fig. 4D, SRTs were generally slower in the overlap condition and this difference was largely unaffected by the target-distractor separation. According to the suppression theory, deviation towards distractors should be observed in the gap condition while deviation away should be observed in the overlap condition, regardless of the target-distractor distance. However, as shown in Fig. 4A and 4B, when the distractor was distal to the target (>90°), the amount of deviation was statistically equivalent for the gap (short SRT) and overlap (long SRT) conditions, see also [49].

## Experiment 2: Stimulus eccentricity

In Experiment 1, the strength of the fixation-related inhibition was manipulated by introducing a temporal gap/overlap between fixation stimulus offset and target/distractor onset. As can be easily inferred from Fig. 3A and 3B, the strength of fixation-related inhibition can also be manipulated by varying stimulus eccentricity. As the eccentricity of the target and distractor increases, the fixation-related inhibition first increases and then decreases. Consequently, when the collicular distance between the target and distractor is fixed, the strength of saccade trajectory deviation in the “away” direction should also increase and then decrease with stimulus eccentricity. This prediction was tested in Experiment 2.

### Method

The human research protocol reported here was approved by the local ethical committee at Hangzhou Normal University and all participants gave written informed consent.

#### Participants

Sixteen university students (10 female, 6 male) participated this experiment for monetary compensation (40 ¥/hour). They all reported having normal or corrected to normal vision and their mean age was 23 years.

#### Stimuli, apparatus and procedure

All stimuli were presented against a black background on a 19 inch CRT monitor. The visible area of the monitor measured 33.5°×25° at a view distance of 62 cm. Stimulus presentation and data collection was controlled by a Mac Mini running custom software written in Python. An Eyelink 1000 (desktop mount) eye tracker, with a spatial resolution of 0.2° or better and a sampling rate of 500 Hz, was used to record the participant's direction of gaze.

The task procedure was similar to that of Experiment 1. Self-paced drift correction was performed at the beginning of each trial, followed by the presentation of a gray fixation cross (1°×1°) and then the saccade target. The saccade target was a filled gray square and the distractor was a gray circle. To compare saccade trajectory deviations across multiple eccentricities, it is important to keep the size of the target and distractor and the distance between them comparable across different eccentricities in collicular space. The stimulus eccentricity was 4°, 12° or 24° (visual angle) in Experiment 2. Based on the mapping functions between the SC and the visual field, as described in [79], the angular separation between the target and distractor was set to 63°, 45° and 40.5° (polar angle) and the dimensions of the stimuli were set to 1°×1°, 2.2°×2.2° and 3.98°×3.98° (visual angle), respectively. To accommodate the condition that involved stimuli of 24° eccentricity in our CRT monitor, the fixation cross was either presented 12° left or right to the center of the display (blocked). The distractor was not presented on 50% of the trials. The target could appear either in the upper or lower visual field while the distractor, when presented, always appeared at a mirror location in the opposite visual field. To discourage anticipatory responses, the target was not presented on 10% of the trials.

The participants were tested with four blocks of 120 trials. A practice block of 20 trials was provided at the beginning of each experimental session. A trial was regarded as an error trial and a warning message was displayed if saccades of amplitudes greater than 1.5° were detected during fixation or the landing position of the critical saccade missed the target for more than 2° (visual angle). All error trials were later presented to the participant in a random order, until all trials were completed correctly.

### Results

Successfully completed trials were further cleansed to improve data quality. Trials were excluded from the analyses if the SRT was shorter than 80 ms or longer than 550 ms (1.72%) or the starting point of the primary saccade deviated more than 2.0° from the fixation cross (4.16%). Based on the criteria given by Van Selst and Jolicoeur [80], a trial was also excluded if the directional deviation from the target direction was above or below 2.35 standard deviations from the mean (2.18%). After data cleansing, 91.17% of the trials remained.

**Figure 5:**
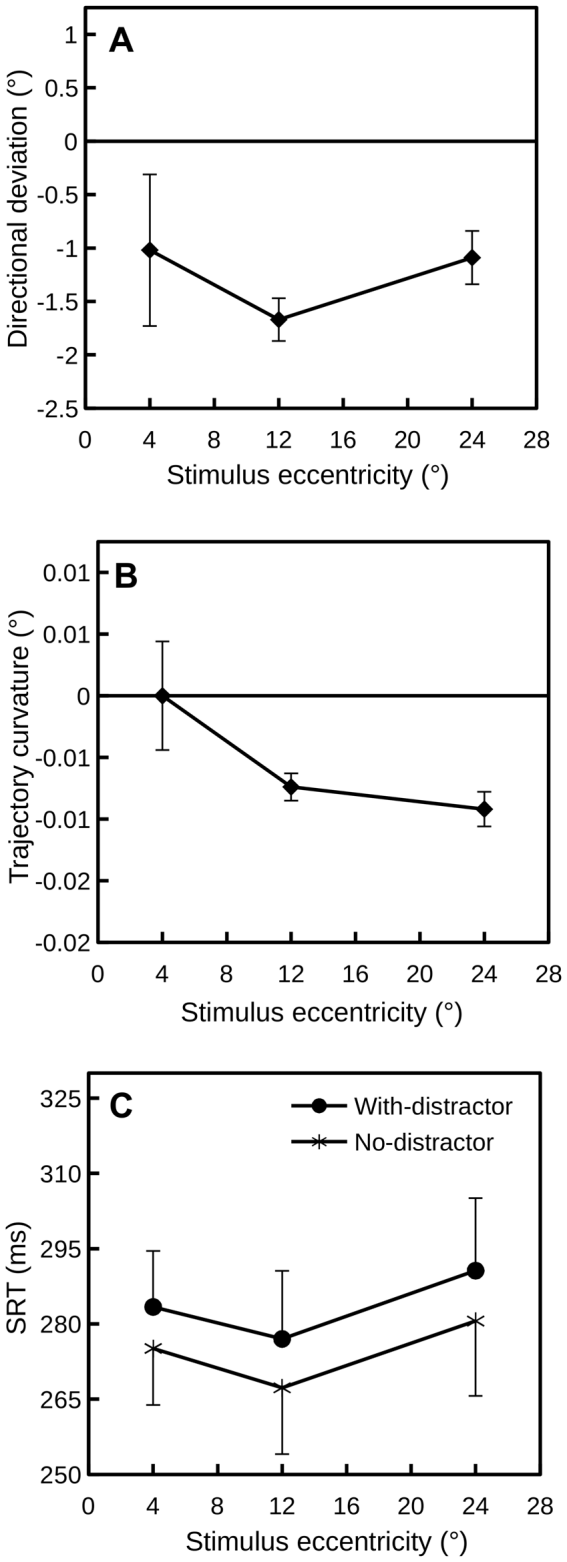
Mean directional deviation (A), trajectory curvature (B), and SRT (C) for each condition in Experiment 2. Error bars denote ±1 SE; for clarity, only one side of the error bars is drawn in (C).

Dependent measures of interest included initial directional deviation, maximum curvature, and SRT. Unlike Experiment 1 where stimulus eccentricity was fixed in all conditions, multiple eccentricity was tested in Experiment 2. To make comparisons across different eccentricities, initial saccade direction was calculated with a sample point where the eyes had travelled approximately 20% of the total duration and maximum curvature was further divided by the amplitude of the saccade. Mean directional deviation, trajectory curvature, and SRT of each condition are presented in Fig. 5.

#### Directional deviation

A single-factor repeated measures ANOVA of directional deviations failed to reveal a main effect of stimulus eccentricity (4°, 12° or 24°), F(2, 30) = 0.98, p = 0.39. However, planned comparisons revealed a reliable difference between the 12° and 24° eccentricities, t(15) = 3.46, p<0.01. Weaker directional deviation was observed for the 24° eccentricity in 13 out of 16 participants. The directional deviation for the 4° eccentricity was numerically weaker than that for the 12° eccentricity. This difference, however, was not statistically reliable, t(15) = 1.23, p = 0.28.

#### Trajectory curvature

Analysis of trajectory curvature revealed a main effect of stimulus eccentricity, F(2, 30) = 3.73, p<0.05. Planned comparisons revealed reliable difference between the 4° and 12° eccentricities, t(15) = 1.89, p = 0.08 (2-tailed), and the 4° and 24° eccentricities, t(15) = 2.01, p = 0.06 (2-tailed).

#### SRT

An ANOVA of the SRTs, with variables distractor presence (with- vs. without-distractor) and stimulus eccentricity, revealed only a significant main effect for distractor presence, F(1, 15) = 18.62, p<0.001. As shown in Fig. 5C, the presence of the distractor prolonged the SRT for about 9 ms across all eccentricities. The main effect of stimulus eccentricity and the two-way interaction did not reach significance.

### Discussion

On the basis of the results of the present experiment, we argue that the strength of the fixation activation in the rostral pole of the SC modulates the strength (and the direction) of deviations of saccade trajectories. As can be easily inferred from Fig. 3B, the effect of the fixation activation first increases and then decreases with stimulus eccentricity. Thus, the theory presented here predicts that the strength of saccade trajectory deviations should vary with stimulus eccentricity, a prediction that was confirmed by Experiment 2. As clearly shown in Fig. 5A, the directional deviation decreased as the stimulus eccentricity increased from 12° to 24°. An increase in directional deviation from 4° to 12° was also observed. This observation, however, was not statistically reliable, because the directional deviation for the 4° eccentricity was highly variable across participants. This variability was likely contributed to by two factors: a) the size of the fixation zone in the SC might be highly variable across participants, and b) the initial saccade direction was estimated with a much earlier gaze sample than that for the 12° and 24° eccentricities.

## General Discussion

Using a distractor paradigm, the present study explored how deviations of saccade trajectory are affected by fixation activity in the SC, with model simulations and behavioral experiments. Three aspects of the model prediction (as presented in Fig. 2C and 4D) were supported by the findings of Experiment 1. First and foremost, saccades deviated towards close distractors and deviated away from remote distractors, see also [49], suggesting that the lateral interactions on oculomotor maps, especially the SC, is a determinant of saccade trajectory deviation. It is important to note that the transition from deviation towards to deviation away from distractors occurred at relatively small target-distractor separations. This observation agrees with previous findings (see Fig. 2C for a summary) and suggests that saccade deviations in humans are better simulated using an interaction kernel of a small excitation zone (see Fig. 3D). Second, there was a general trend for deviation away from distractors to decrease as the target-distractor separation increases, especially in the overlap condition (see Fig. 4A). An ANOVA that only considered target-distractor separations between 45° and 150° revealed a significant main effect target-distractor separation, F(7, 105) = 2.72, p<0.05, which did not interact with condition (gap vs. overlap), F(7, 105) = 1.48, p = 0.18. Most importantly, the amount of saccade trajectory deviation was modulated by the manipulation of temporal gap/overlap (and thus the strength of fixation activity in the SC), regardless of being quantified with directional deviation or trajectory curvature. Confirming the model predictions presented in Fig. 3D, this modulatory effect was strong when the distractor was close to the target and was largely absent when the distractor was distal to the target. Providing further evidence for the model prediction that saccade trajectory deviation is modulated by fixation activity, Experiment 2 show that the strength of directional deviation away from distractors decreases as stimulus eccentricity increased from 12° to 24°.

### Fixation activity and saccade deviation

Saccade latency is usually shortened when the fixation stimulus is removed before target onset, i.e., the gap effect [76]. When the fixation stimulus remains visible after target onset, however, saccade latency is usually prolonged [75]. The manipulation of temporal gap/overlap between fixation offset and target onset has been used in several recent studies to explore the relationship between saccade latency and saccade deviation [6], [18], [19]. One interesting finding of this line of study is that saccade deviation seems to vary with saccade latency (for failure to observe such an effect, see [9], [20]). For instance, McSorley and colleagues [18] demonstrated that short SRTs were accompanied by trajectories curving towards distractors while long SRTs were accompanied by trajectories curving away from distractors. Similar results were also observed in the present behavioral experiment, but only when the target-distractor separation was relatively small (see Fig. 4B). Such observations have been widely regarded as evidence for the suppression theory which argues that suppression at the distractor location takes time to develop. The most significant contribution of the present study is that we show that the not always observed correlation between SRT and saccade deviation is caused by the manipulation of temporal gap/overlap, which in turn affects the strength of fixation activity the SC [70], [71].

### A general explanation for deviation away

The present study and our previous modeling work [1] emphasized the role of the lateral interactions in the SC in generating deviation away from distractors. Here we would like to point out that the lateral interaction in the SC is just one implementation of a much more general principle underpinning deviation of saccade direction. This general principle is non-uniform inhibition or suppression of target activation (see Fig. 2A and Fig. 3C). In addition to the Mexican-hat shaped lateral interaction which could give rise to a non-uniform inhibition from the distractor, previous studies have identified several factors that may cause non-uniform inhibition/suppression of target activation. These factors include, but are not limited to, pharmacological inactivation of the SC [22], [23] and inhibition of return (IOR) [55], [81]. When an SC site is inactivated, there will be a gradient of suppression surrounding the infusion site. When a target appears at a location close to the response field of the infused SC site, due to uneven suppression, saccades will be pushed away (see Fig. 3C). Rather than using pharmacological methods, in a recent study, we successfully produced one such uneven (Gaussian shaped) inhibition behaviorally, taking advantage of a behavioral phenomenon called IOR [82]–[84], see also [6]. When a target is presented close to a recently visually stimulated location, response times (RTs) are usually slower than when the same target appears at a location that has not been stimulated. This RT cost has a spatial gradient, as the distance between the stimulated location and the target increases RT decreases [85], [86]. When the angular separation between the stimulated location and the target was varied, saccades deviated away from the stimulated location even when the separation was only 15° [81]. In contrast to distractor induced deviation away which could only emerge when the distractor is relatively distal to the target (>30°; see Fig. 2C and Fig. 4A), IOR or inactivation can induce deviation away when the saccade target is presented very close to the location suppressed by IOR [81] or inactivation [22]. It should be noted that there is abundant evidence supporting the notion that IOR mainly affects the visual pathway [25], [87]–[89]. Considering the fact that inactivation directly suppresses neuronal activity in the SC, it is safe to conclude that deviation away can be caused by *non-uniform suppression of target activation* at various stages of processing. This general theory is in contrast to the suppression theory which emphasizes the *suppression of distractor activation*.
